# Primary ovarian abscess in virginal young woman with huge endometriosis cyst: A case report

**DOI:** 10.1097/MD.0000000000029463

**Published:** 2022-05-27

**Authors:** Wenhua Liu, Zhifen Zhang, Dinghen Li

**Affiliations:** Department of Obstetrics and Gynecology, Hangzhou Women's Hospital (Hangzhou Maternity and Child Health Care Hospital), Hangzhou, China.

**Keywords:** endometriosis cyst, primary ovarian abscess, virginal young woman

## Abstract

**Rationale:**

Primary ovarian abscess which develops as an isolated lesion without simultaneous tubal infection is a rare entity. Ovarian abscess (OA) is a serious complication of pelvic inflammatory disease (PID) rarely seen in virginal girls. Early diagnosis and treatment are essential to prevent further sequela including infertility, ectopic pregnancy, and chronic pelvic pain.

**Patient concerns:**

A 19-year-old virginal girl who presented with abdominal pain and pelvic mass with no risk factors.

**Diagnoses:**

Laparoscopic surgery was performed to confirm a primary ovarian abscess in the adolescent virginal female with a huge endometriosis cyst.

**Intervention:**

Ovarian abscess with extensive intestinal adhesions was determined during the laparoscopic operation. Abscess drainage and postoperative antibiotic therapy cured the patient.

**Outcome:**

After the surgery, the CRP level on the day of discharge was 3.18 mg/d. The histological findings revealed a cystic tissue sample with the fibrous wall infiltrated by neutrophilic granulocytes, and ectopic endometrium, suggesting abscess formation in the ovary and endometriosis cyst.

**Lessons:**

Although primary ovarian abscess in an adolescent virginal female is rare, given the severity of outcomes following ovarian abscess, this pathology should be considered in the differential diagnosis of virginal adolescents with fever and abdominal pain.

## 1 Introduction

Primary ovarian abscess is defined as ovarian infection without tubal involvement, is a rare entity. Furthermore, Ovarian abscess most often occurs in sexually active women, which is extremely rare in virginal adolescent females.^[1]^ Endometriosis cyst is a common disease. However, the collection of altered menstrual-type blood in a cystic space in the ovary can be a suitable culture medium for pathogens. Pathogenic organisms are generally introduced into an ovarian endometrial cyst after surgical drainage or transvaginal aspiration^.^^[2]^

We present a unique case of primary ovarian abscess with a huge endometrial cyst in virginal young women with no risk factors.

## 2 Case

A 19-year-old healthy virginal young woman was referred to the obstetrics and gynecology hospital by a local doctor after she presented with persistent fever (up to 39 °C for a month) and chronic abdominal pain for a month. The patient denied any recent infection or sexual activity and her medical history was normal. Her abdomen was stiff and she had involuntary guarding and rebound tenderness in the lower right quadrant. A pelvic exam was not performed as she was virginal. Laboratory data showed a white blood cell (WBC) count of over 11.1 × 10^9^/L and a C-reactive protein (CRP) level of 83.36 mg/L. Additionally, assays for procalcitonin (PCT), Alpha-fetoprotein (AFP), carbohydrate antigen 19-9 (CA 19-9), cancer antigen 15-3 (CA 15-3), carcinoembryonic antigen (CEA), carbohydrate antigen 125 (CA-125), and human epididymal protein 4 (HE4), respectively, were negative.

**Figure 1:**
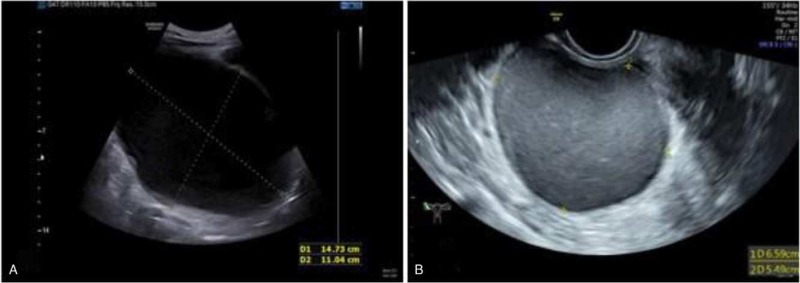
Abdominal ultrasound notes an anechoic black area measuring 6 cm, considering an endometriosis cyst a month ago (A), a huge pelvic mass measuring 15 cm at the patient's second visit (B).

The transabdominal ultrasound showed an anechoic black area of 6 cm, considering an endometriosis cyst a month ago. On the patient's second visit, a transabdominal ultrasound was performed, which revealed a huge pelvic mass of 15 cm in the right adnexal area (Fig. 1).

**Figure 2:**
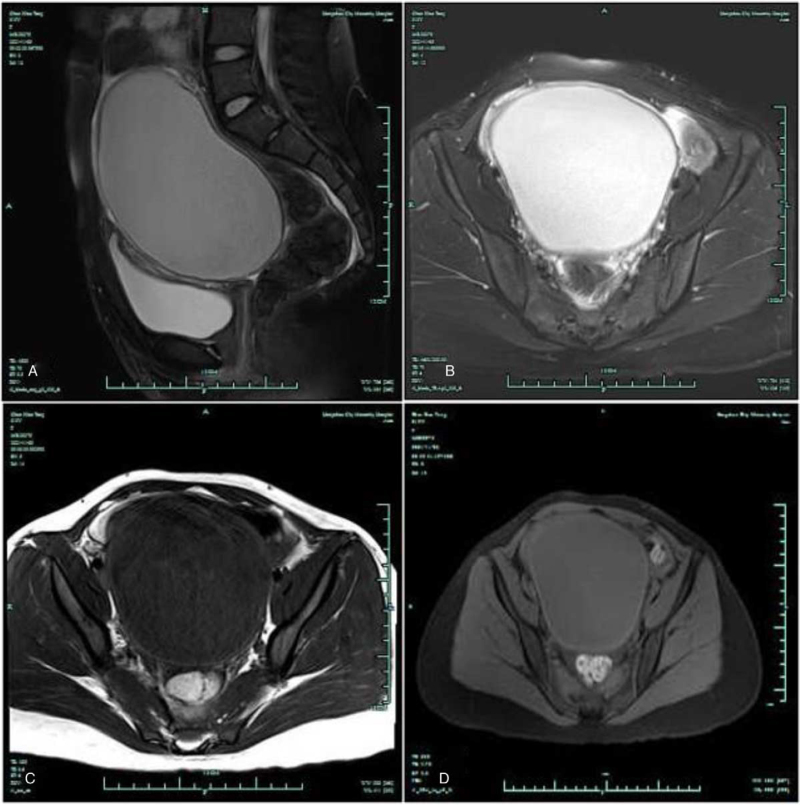
Magnetic resonance imaging in a sagittal image (A), a right ovarian mass with high signal intensity on T2-weighted images (B), low signal intensity on T1-weighted images (C), iso-hyper signal intensity on diffusion-weighted imaging (D). The white arrow shows the huge pelvic mass.

Magnetic resonance imaging (MRI) showed high signal intensity in T2-weighted images, low signal intensity in T1-weighted images, and iso-hyper signal intensity in diffusion-weighted imaging. This observation suggested a similar ovarian tumor (Fig. 2).

**Figure 3:**
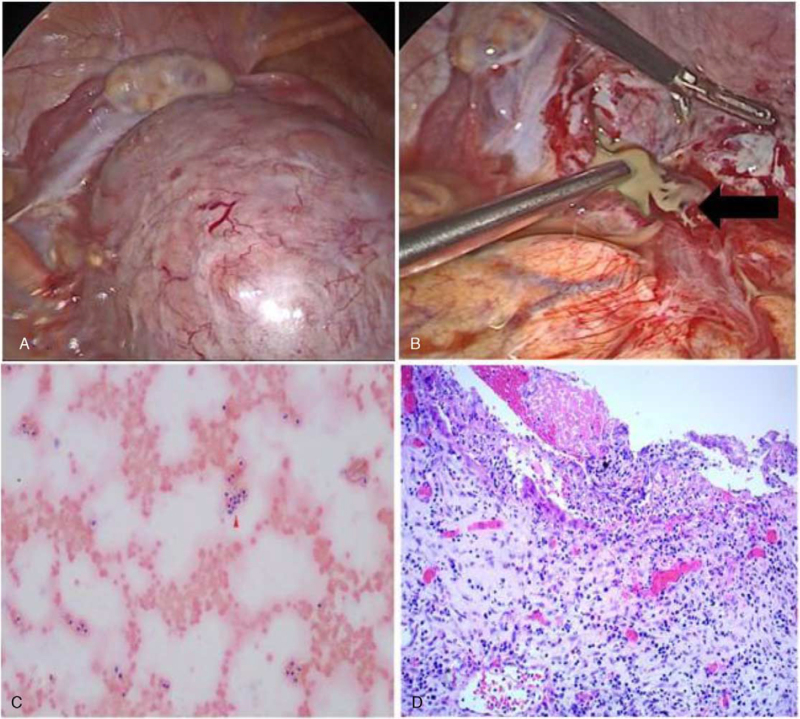
Laparoscopy shows unruptured right ovarian abscess with extensive omental and bowel adhesions. The right ovarian abscess with intact fallopian tube and intact left ovary with intact fallopian tube before excision (A) and during excision (B). The black arrow shows the abscess. Massive inflammatory cells in the peritoneal wash were found (C). The histological findings revealed a cystic tissue sample with the fibrous wall with neutrophilic infiltration, ectopic endometrium, suggesting abscess formation in the ovary (D).

Given the risk of ovarian abscess rupture and acute sepsis due to persistent fever, laparoscopic surgery was performed to confirm and excise the abscess and the pelvic mass was determined to be an unruptured right ovarian abscess with extensive omental and bowel adhesions. Laparoscopy revealed a 15 cm swollen right ovary with an intact fallopian tube (Fig. 3); an intact left ovary with an intact fallopian tube; and a small amount of ascites. Puncturing of the swollen right ovary revealed internal pus, which confirmed the diagnosis of OA. The pus was collected for bacterial culture and the abscess was excised without any substantial compromise to the ovary (Fig. 3B). No bacteria were cultured from the pus. We performed pelvic washing and pelvic exenteration. The postoperative laboratory data 6 ays after surgery showed a CRP level of 20.27 mg/d. The patient received intravenous cefoperazone/sulbactam for 5 days at a dose of 2 g/day to prevent the recurrence of the infection. The postoperative course was uneventful, and the patient was discharged 5 days after surgery; the CRP level on the day of discharge was 3.18 mg/d. Massive inflammatory cells in the peritoneal wash were found (Fig. 3C). The histological findings revealed a cystic tissue sample with the fibrous wall infiltrated by neutrophilic granulocytes, ectopic endometrium, suggesting abscess formation in the ovary and endometriosis cyst (Fig. 3D).

## 3 Discussion

Ovarian infection without tubal involvement, i.e. primary ovarian abscess, is extremely rare in virginal adolescent women.^[3]^ Approximately 85% of cases are infections in sexually active females of the reproductive age group. Pelvic abscess and PID are most often sequelae of a sexually transmitted disease and it is very rarely reported in virgins. A review of the literature revealed more than 10 cases of tubo-ovarian abscesses (TOA) or OA in sexually inactive girls.^[4]^ The cause of TOA or OA in this patient group is often unclear. However, virginal girls have been speculated to have comorbidities, such as vaginal voiding causing ascending infection, gastrointestinal tract translocation, congenital genitourinary anomalies, previous pelvic surgery, and bacteremia from skin wounds, which predispose them to OA or TOA.^[4,5]^ The present case was with no unusual risk factors. Therefore, practicing clinicians should consider the possibility of TOA or OA in adolescent females presenting with abdominal pain and adnexal mass regardless of their sexual activity, especially in the absence of risk factors.

Bacteria were not examined in the bacterial culture of the abscess in this case. The possible reason was that specimen was not examined timely. In the present case, since the fallopian tubes were intact and the patient had no episode of transvaginal maneuver, the source of the infection could have been via the bloodstream. Moreover, the patient had no history of dental treatment, trauma, or compromised immune system; therefore, no alternative source of infection was identified. Thus, the present case suggests that clinicians should consider the possibility of uncommon bacterial species as the causative agent for OA among virginal girls and that these species may cause infection from an unknown origin via the bloodstream.

PID and ovarian abscess occur more frequently and are more severe in women with endometriosis than in those without endometriosis.^[6]^ Endometriosis can aggravate tubal adhesions and distortions through intrinsic pathological mechanisms such as inflammatory microenvironment independent of disease type or severity.^[7]^ There are two main theories to explain the reason, one is the bloody content of the endometrioma or in the peritoneal cavity. The other possible reason may be immune system dysfunction.^[8]^ The characteristics and treatment of TOA patients with endometriosis are similar to those without endometriosis. However, endometriosis more often generates serious complications and surgical bleeding in TOA patients.^[8]^ Pelvic inflammatory disease in women with endometriosis is more severe and refractory to antibiotic treatment, often requiring surgical intervention.^[9,10]^ Our present case showed an ovarian abscess with extensive bowel adhesions. Therefore, we performed the removal of a section of cyst wall, pelvic washing, and pelvic exenteration.

## 4 Conclusions

In summary, we present a unique case of a primary ovarian abscess in the adolescent virginal female with a huge endometriosis cyst. Although primary ovarian abscess in an adolescent virginal female is rare, given the severity of outcomes following ovarian abscess, this pathology should be considered in the differential diagnosis of virginal adolescents with fever and abdominal pain. Additionally, endometriosis can aggravate the severity of OA.

## Acknowledgment

The authors thank the patient who made this work possible and gratefully acknowledge the support of the clinicians and researchers who contributed to this study. The informed consent was obtained from the patient.

## Author contributions

Wenhua Liu and Dinghen Li contributed to the conception, design, data collection, statistical analysis, and drafting of the manuscript. Zhifen Zhang contributed to the preliminary review. All authors have seen and approved the final manuscript.

**Data curation:** Wenhua liu.

**Supervision:** Dinghen Li, Zhifen zhang.

**Writing – original draft:** Wenhua liu.

**Writing – review & editing:** Wenhua liu.
